# Structural covariance networks are coupled to expression of genes enriched in supragranular layers of the human cortex

**DOI:** 10.1016/j.neuroimage.2017.12.060

**Published:** 2018-05-01

**Authors:** Rafael Romero-Garcia, Kirstie J. Whitaker, František Váša, Jakob Seidlitz, Maxwell Shinn, Peter Fonagy, Raymond J. Dolan, Peter B. Jones, Ian M. Goodyer, the NSPN Consortium, Edward T. Bullmore, Petra E. Vértes

**Affiliations:** a Department of Psychiatry, University of Cambridge, Cambridge, CB2 0SZ, UK; b The Alan Turing Institute for Data Science, British Library, 96 Euston Road, London, NW1 2DB, United Kingdom; c Research Department of Clinical, Educational and Health Psychology, University College London, London, WC1E 6BT, UK; d Wellcome Trust Centre for Neuroimaging, UCL Institute of Neurology, University College London, London, WC1N 3BG, UK; e Max Planck UCL Centre for Computational Psychiatry and Ageing Research, London, WC1B 5EH, UK; f Cambridgeshire and Peterborough NHS Foundation Trust, Huntingdon, PE29 3RJ, UK; g Department of Psychiatry, University of Cambridge, Cambridge, CB2 0SZ, UK; h Cambridgeshire and Peterborough NHS Foundation Trust, Huntingdon, PE29 3RJ, UK; i ImmunoPsychiatry, Immuno-Inflammation Therapeutic Area Unit, GlaxoSmithKline R&D, Stevenage, SG1 2NY, UK

**Keywords:** Allen Human Brain Atlas, Cortical thickness, Gene expression, Structural brain network, Transcriptomic brain network

## Abstract

Complex network topology is characteristic of many biological systems, including anatomical and functional brain networks (connectomes). Here, we first constructed a structural covariance network from MRI measures of cortical thickness on 296 healthy volunteers, aged 14–24 years. Next, we designed a new algorithm for matching sample locations from the Allen Brain Atlas to the nodes of the SCN. Subsequently we used this to define, transcriptomic brain networks by estimating gene co-expression between pairs of cortical regions. Finally, we explored the hypothesis that transcriptional networks and structural MRI connectomes are coupled.

A transcriptional brain network (TBN) and a structural covariance network (SCN) were correlated across connection weights and showed qualitatively similar complex topological properties: assortativity, small-worldness, modularity, and a rich-club. In both networks, the weight of an edge was inversely related to the anatomical (Euclidean) distance between regions. There were differences between networks in degree and distance distributions: the transcriptional network had a less fat-tailed degree distribution and a less positively skewed distance distribution than the SCN. However, cortical areas connected to each other within modules of the SCN had significantly higher levels of whole genome co-expression than expected by chance.

Nodes connected in the SCN had especially high levels of expression and co-expression of a human supragranular enriched (HSE) gene set that has been specifically located to supragranular layers of human cerebral cortex and is known to be important for large-scale, long-distance cortico-cortical connectivity. This coupling of brain transcriptome and connectome topologies was largely but not entirely accounted for by the common constraint of physical distance on both networks.

## Highlights

• Transcriptomic Brain Network (TBN) is defined as inter-regional gene co-expression.
• TBN has complex topological properties partially overlapped with structural networks.
• Structural modules have higher gene co-expression than expected by chance.
• Human Supragranular genes are highly expressed and coexpressed in structural hubs.

## Introduction

Many natural systems have a complex network topology. Nervous systems form anatomical networks with graph-theoretically non-random properties like small-worldness, high-degree hubs and modules (Bullmore and Sporns, 2009). Brain networks conserve these topological features from the micro (synaptic) scale of *C. elegans* to the macro scale of whole human brain connectomes derived from statistical analysis of human magnetic resonance imaging (MRI) data (Fornito et al., 2016). A similar topological profile has also been described for networks of gene expression, in which each node is a gene and each edge represents the strength of correlation or co-expression of genes between samples collected in different tissue types or across different individuals or different time-points (Belcastro et al., 2011).

Here we define a brain network whose nodes represent brain regions and whose links are based on pairwise correlations in whole-genome gene expression across brain regions. We then investigate the relationship between this transcriptional brain network (TBN), or transcriptome, and a structural covariance network (SCN), or connectome, constructed from MRI data on 296 healthy participants.

A structural covariance network describes the existence of positively correlated brain regional anatomical measurements – such as cortical thickness or volume – between pairs of brain regions (Wright et al., 1999). Structural covariance networks are replicable, heritable, and representative of disease-related changes in topology (Alexander-Bloch et al., 2013). However, the neurobiological substrate of inter-regional structural covariation remains poorly understood. There is some evidence that structural correlation is related to anatomical connectivity between regions; but MRI networks based on inter-regional correlation of cortical thickness (CT) are only moderately similar to diffusion tensor imaging (DTI) networks based on tractographic analysis of white matter projections between cortical areas (Gong et al., 2012).

Genotype has a major influence on structural features of the human cortex such us areal expansion (Chen et al., 2013) and cortical thickness (Fjell et al., 2015). Moreover, brain regions sharing high genetic covariance are known to have a complex network organization (Docherty et al., 2015). In addition to genotype, transcriptomic spatiotemporal profiles also have a strong impact on neurodevelopmental processes (Wong et al., 2017), cell types (Kang et al., 2011) and cognitive specialization (Johnson et al., 2009). For example, recent studies of the mouse brain have revealed that structurally covariant regions (Fulcher and Fornito, 2016) and highly connected nodes (Yee et al., 2017) have similar transcriptional profiles. However, the role of transcriptomic similarities in the morphological features of the human cortex remains unexplored.

Gene expression in all regions of human cortex can be estimated using the transcriptomic dataset made publicly available by the Allen Institute for Brain Science (AIBS). The AIBS used microarray data to produce the first comprehensive genome-wide atlas of gene expression in the brain that can be mapped into MNI anatomical space (Hawrylycz et al., 2012). Previous analysis of these data has demonstrated that the transcriptional profile of cortical tissue is fairly homogenous compared to variations between cortex and subcortex (Hawrylycz et al., 2012). However, cortical gene expression has been shown to correlate with resting state functional connectivity (Hawrylycz et al., 2015), revealing a strong association with genes enriched for ion channel and synaptic ontology terms (Hawrylycz et al., 2015, Richiardi et al., 2015). Functional connectivity has also been specifically related to expression of a set of human supragranular expressed (HSE) genes (Krienen et al., 2016, Vértes et al., 2016) that are highly expressed in the supragranular layers of human cortex (lamina 2, 3), where most cortico-cortical projections originate (Zeng et al., 2012). Nonetheless, the coupling between the co-expression of HSE genes and the cortico-cortical covariation in nodal structure defined by the SCN has not previously been assessed. Moreover, HSE genes have a differential expression signature across cell types that delineate the cytoarchitectural boundaries of the cortex (Zeng et al., 2012). Although previous studies have explored the association between cortico-cortical connectivity and the underlying cytoarchitectonics (Beul et al., 2015), the transcriptomic similarity between cytoarchitectonic classes for the complete human cortex has not been established.

Here we explored the hypothesis that putative metrics of anatomical connectivity of the human cortex – such as inter-regional correlations of cortical thickness – should be related to gene co-expression, and specifically to co-expression of HSE genes. In what follows, we first develop a new method for matching samples from the AIBS to MRI regions of interest in the native space of each postmortem AIBS brain (AIBS postmortem data available at http://human.brain-map.org/). On this basis we construct a transcriptional brain network (TBN) and describe, for the first time, its properties as a complex network using graph theoretical analysis, as well as its similarities with the structural covariance network (SCN; connectome). Additionally, we assess the transcriptional similarities between regions with the same cytoarchitecture based on the Von Economo atlas (von Economo, 1929), which divides the cortex according to laminar structure. Finally, we explored the relationship between the two networks, testing the specific hypotheses: (i) that regions connected as part of the same modules of the SCN have higher levels of whole genome and HSE gene co-expression than regions in different modules; (ii) that highly connected regions overexpress HSE genes; (iii) that regions connected in the SCN have higher levels of HSE gene co-expression than regional nodes that were not anatomically connected; and (iv) that the relationships between structural correlation and gene co-expression were not entirely attributable to common constraints of physical connection distance on both these spatially embedded networks.

## Methods

### Participants

2135 healthy young people in the age range 14–25 years were recruited from schools, colleges, NHS primary care services and direct advertisement in north London and Cambridgeshire. This primary cohort was stratified into 5 contiguous age-related strata: 14–15 years inclusive, 16–17 years, 18–19 years, 20–21 years, and 22–25 years. Recruitment within each stratum was evenly balanced for sex and ethnicity and satisfied exclusion criteria including any history of treatment for psychiatric disorder or drug or alcohol dependence, or any history of neurological disorder, head injury or learning disability. A demographically balanced cohort of 300 participants was sub-sampled from the primary cohort for structural MRI scanning in one of the following sites: (1) Wellcome Trust Centre for Neuroimaging, London; (2) Medical Research Council Cognition and Brain Sciences Unit and (3) Wolfson Brain Imaging Centre, Cambridge.

Written informed consent was provided by all participants as well as written parental assent for participants less than 16 years old. The study was ethically approved by the National Research Ethics Service and was conducted in accordance with NHS research governance standards.

### MRI data acquisition

Structural MRI scans were acquired in three different locations (two in Cambridge and one in London) using identical 3T MRI systems (Magnetom TIM Trio, Siemens Healthcare, Erlangen, Germany; VB17 software version). The setup, acquisition and post-processing were previously described (Weiskopf et al., 2013). Briefly, the multi-parametric mapping (MPM) protocol yields 3 multi-echo fast low angle shot (FLASH) scans with different predominant weightings: T1 (TR = 18.7 ms, α = 20°), Proton Density (PD) or Magnetization Transfer (MT) (TR = 23.7 ms, α = 6°). Multiple gradient echoes were generated for each FLASH acquisition with alternating readout polarity echo times (TE): (i) for T1-weighted (T1w) and MT-weighted (MTw) 6 six equidistant acquisitions between 2.2 and 14.7 ms and (ii) for the PD-weighted (PDw) 8 equidistant acquisition with TE between 2.2 ms and 19.7 ms. By applying previously developed models describing the image intensity of FLASH scans (Helms et al., 2008a, Helms et al., 2008b, Weiskopf et al., 2011) to the resulting T1w, MTw and PDw images, we calculated the parameter maps of the apparent longitudinal relaxation rate R1 (R1 = 1/T1), the MT saturation, and the effective proton density PD*. Here, only R1 quantitative maps were used. Apparent R1 maps were corrected for local RF transmit field inhomogeneities and imperfect RF spoiling (Preibisch and Deichmann, 2009) to create quantitative R1 maps (Weiskopf et al., 2013).

Other acquisition parameters were: 1 mm^3^ voxel resolution, field of view (FOV) = 256 × 240 mm, 176 sagittal slices, parallel imaging using GRAPPA factor 2 in anterior-posterior phase-encoding (PE) direction, RF spoiling phase increment = 50°, 6/8 partial Fourier in partition direction, non-selective RF excitation, readout bandwidth BW = 425 Hz/pixel. A pilot study demonstrated satisfactory levels of between-site reliability in MPM data acquisition (Weiskopf et al., 2013).

### MRI reconstruction, cortical parcellation and SCN construction

We used a standard automated processing pipeline (FreeSurfer v5.3) for skull stripping, tissue classification, surface extraction and cortical parcellation (http://surfer.nmr.mgn.harvard.edu) applied to longitudinal relaxation rate (R1) maps. All scans were quality controlled by experienced researchers to reduce the impact of motion artifacts and improve cortical thickness estimation. Freesurfer reconstructions were visually inspected and manually edited up to 10 times. At each iteration, control points and grey/white matter edits were included and Freesurfer recon-all reconstruction was repeated. Only those cortical reconstructions that finished without error were included in the analyses. 4 out of the 300 MRI scans collected were excluded because they did not meet these QC requirements, resulting in a final MRI sample of 296 subjects (19.11 ± 2.93 years [mean ± standard deviation], 148 females).

**Fig. 1:**
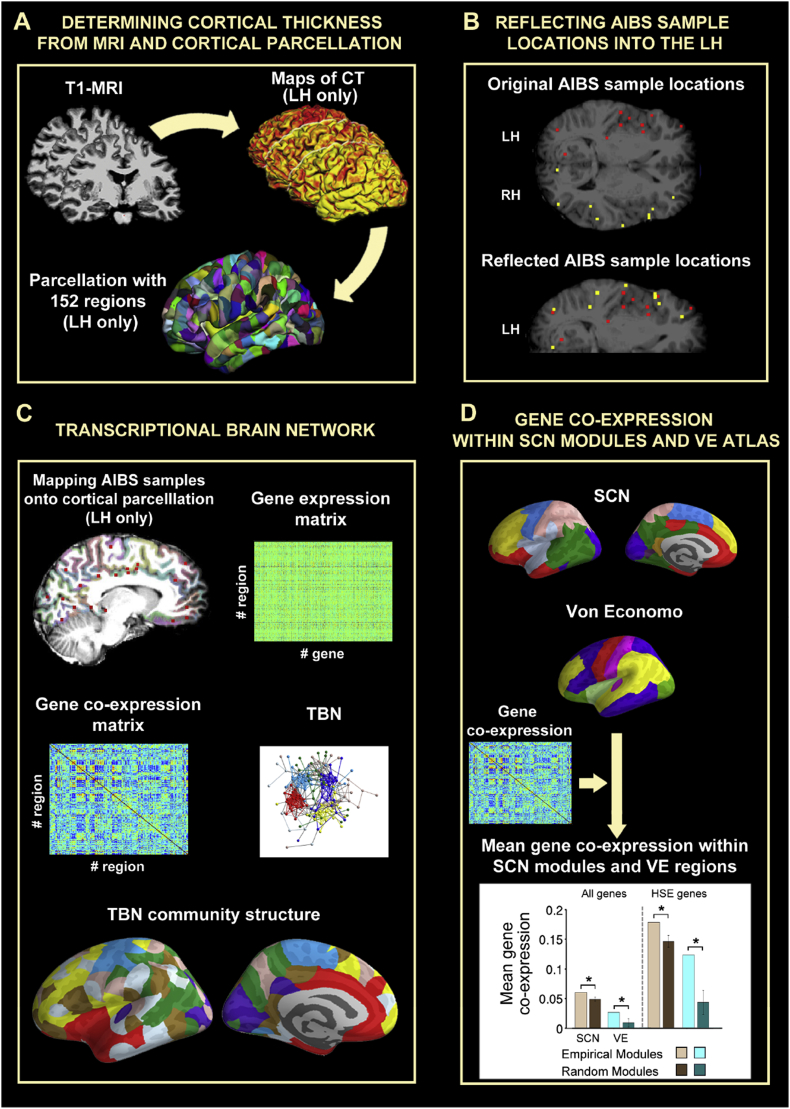
A schematic overview of data analysis. **A:** Cortical thickness was estimated from R1-weighted MRI scans on 296 healthy young participants. The left hemisphere (LH) was sub-divided into regional nodes by a fine-grained parcellation of 152 nodes based on the Desikan-Killiany atlas. **B:** Reflection of AIBS gene expression profiles sampled in the right hemisphere (RH, yellow dots) onto the LH (red dots) to increase sample density. **C:** Regional gene expression profiles from the AIBS Human Brain Atlas were mapped to the same cortical parcellation template to estimate regional expression profiles and a co-expression matrix. The gene expression matrix was thresholded to construct a binary graph which had a modular community structure. **D:** Modules of the structural covariance network, and cytoarchitectonic classes defined by von Economo, were used to test the hypothesis that whole genome co-expression and HSE gene co-expression were greater between nodes in the same topological module or cytoarchitectonic class.

Cortical thickness (CT) measurements were estimated by reconstructing the pial surface and the boundary between grey matter and white matter (Dale and Sereno, 1993, Dale et al., 1999) and measuring the distance between these surfaces; see Fig. 1A.

Due to under-sampling of the right cerebral hemisphere in the gene expression data (see below; Hawrylycz et al., 2012) we focused attention on the left hemisphere of both the MRI and gene expression data. We used a high-resolution parcellation of the left hemisphere that comprised 152 cortical regions, each with an approximate surface area of 500 mm^2^. Consequently, MRI data corresponding to the right hemisphere were discarded. The cortical parcellation of the left hemisphere was created in FreeSurfer template (fsaverage) space by sub-dividing the anatomically defined regions in the Desikan-Killiany atlas (Desikan et al., 2006) (Romero-Garcia et al., 2012); see Fig. 1A. R1-weighted images of each subject were co-registered to the FreeSurfer surface template using rigid transformations to obtain the transformation matrix $T_{R1}$. Inverse transformations ($T_{R1}^{-1}$) were used to warp the parcellation scheme from standard stereotactic space to the space of each individual R1-weighted image. CT values were averaged across all vertices included in each cortical parcel. This process was repeated for each subject resulting in a (296 × 152) matrix of cortical thickness estimates at each of 152 regions for each of 296 participants.

The structural covariance matrix of a group of subjects is defined by estimating the inter-regional correlation of cortical thickness between all possible pairs of regions defined by an anatomical parcellation (He et al., 2007). Thus, SCN construction relies on the identification of spatial patterns of morphometric similarities between brain regions within a group of subjects. The inter-regional correlations in cortical thickness were estimated to construct a (152 × 152) structural correlation matrix. A hard threshold was applied to this matrix so that an arbitrary percentage (also called connection density) of the most strongly positive CT correlations were retained as non-zero elements in a binary adjacency matrix or, equivalently, edges between regional nodes in a graph of the structural covariance network. Brain networks were visualized using BrainNet viewer (Xia et al., 2013) (http://www.nitrc.org/projects/bnv/).

### AIBS gene expression dataset

We used the whole genome expression atlas of the adult human brain created by the Allen Institute for Brain Science (http://human.brain-map.org) (Hawrylycz et al., 2015, Hawrylycz et al., 2012). The AIBS dataset includes samples from post-mortem brains of six donors (3 Caucasian, 2 African-American, 1 Hispanic) aged 24–57 years. Custom 64K Agilent microarrays were used to measure expression of all genes in the genome at several hundred cortical and subcortical locations in each donor's brain. Given the strong expression differences between subcortical and cortical tissue, the present study only includes samples labeled as “Cortical” by the AIBS. As a consequence, no subcortical samples were used to estimate the gene expression of cortical parcels even if subcortical genomic samples intersected cortical parcels due to registration misalignment of the AIBS and MRI datasets (Hawrylycz et al., 2012). According to AIBS annotation, 48171 out of 58692 probes can be mapped onto genes. Here, we used Richiardi et al.*’s* (2015) criterion to reannotate the probe sequences using the genome assembly hg19 (UCSC Genome Browser; http://sourceforge.net/projects/reannotator/; (Arloth et al., 2015)). After the reannotation a final set of 49770 probes was associated with genes. For genes that were mapped by multiple cRNA hybridization probes, the probe showing highest average expression across samples was selected. Previous studies have shown the high between-study consistency of this probe-to-gene mapping method (Miller et al., 2011). As a result, 20,647 gene expression values were evaluated in 3702 brain samples. As AIBS only sample two of the six donors in the right hemisphere and given the strong expression similarities between hemispheres and in order to increase the number of gene expression samples per cortical region, all right hemisphere samples were reflected to their contralateral position in the left hemisphere (Fig. 1B). Consequently, the final gene expression profile of each region was estimated as the mean of both ipsilateral samples (from all six donors whose left hemisphere was sampled) and contralateral samples (from the two donors whose right hemisphere was also sampled).

### Matching MRI parcellation with sample locations

We developed a new method to map the anatomical structure associated to each tissue sample by using the MRI data provided by the AIBS for each donor. T1-images of the six donors were processed following the FreeSurfer pipeline. The high-resolution parcellation with 152 cortical regions in the left hemisphere, used in the analysis of the NSPN MRI data, was warped from fsaverage space to the surface reconstruction of each AIBS donor's brain. The surface-based parcellation of each donor's brain was transformed into a volumetric parcellation that covered the whole cortex and extended 2 mm into the subjacent white matter to include those cortical samples that had been excluded due to registration misalignment; Fig. 1C. 91% of the total AIBS gene expression cortical samples were located within the resulting volumetric parcels. The matching algorithm excluded 210 out of 1802 samples that were located in the cortex according to their MNI coordinates because they did not fall into any parcel in the corresponding donor's brain reconstruction. On the other hand, 166 samples that did not fall into any parcel in the MNI-152 1 mm brain were associated with a cortical region when the corresponding donor's brain was considered. As a result of this matching algorithm based on individual donor's brains, 62% of the samples were re-annotated to a close-by but different brain parcel compared with the matching based on the MNI-152 brain. Sample reannotations were particularly relevant for the high-resolution parcellation defining cortical regions with small areas. The proportion of reannotated samples was smaller (46%) when the data were parcellated by the more coarse-grained Desikan-Killiany atlas.

In the high resolution parcellation used here, gene expression had to be linearly interpolated for one parcel that contained zero AIBS samples (inferior supramarginal gyrus). Figure S1 shows the number of AIBS samples covered by each cortical region. Gene expression values for each cortical parcel were estimated as the median normalized gene expression over all six donors and compiled to form a (152 × 20,647) matrix representing the expression of each of 20,647 genes at each of 152 left hemisphere cortical regions; Fig. 1C. Code used to estimate these gene expression levels can be downloaded here: https://github.com/RafaelRomeroGarcia/geneExpression_Repository and Freesurfer reconstructions of each donor's brain are available at the Cambridge Data Repository (Romero-Garcia et al., 2017).

### Transcriptional brain network construction

The (152 × 152) gene co-expression matrix was estimated by the pairwise Pearson's correlations between whole genome expression profiles in each possible pair of cortical regions (Fig. 1C). A hard threshold was applied to this matrix so that an arbitrary percentage (also called the connection density) of the most strongly positive transcriptional correlations was retained as non-zero elements in a binary adjacency matrix or, equivalently, as edges between regional nodes in a graph of the transcriptional brain network (TBN).

### Human supragranular enriched (HSE) genes and gene ontologies

The human supragranular enriched (HSE) gene list comprises 19 genes: *BEND5, C1QL2, CACNA1E, COL24A1, COL6A1, CRYM, KCNC3, KCNH4, LGALS1, MFGE8, NEFH, PRSS12, SCN3B, SCN4B, SNCG, SV2C, SYT2, TPBG* and *VAMP1* (Zeng et al., 2012). The z-score normalized expression profile of each HSE gene in each of 152 cortical regions was extracted from the whole genome transcription matrix. The 19 normalized gene expression values were averaged to create an HSE gene expression index. Coupling between SCN and HSE co-expression was compared with gene co-expression of the whole genome and with the co-expression of each of the 5917 gene sets defined in the Molecular Signatures Database version 6.0 (MSigDB) collection (Liberzon et al., 2011). This collection includes gene ontologies for biological processes (4436 gene sets), cellular components (580 gene sets) and molecular functions (901 gene sets).

### Network analysis and community structure

Global topological properties of the structural correlation and transcriptional networks were analyzed using the following graph theoretical measures: clustering coefficient ($C_{p}$), path length ($L_{p}$), local efficiency ($E_{loc}$), global efficiency ($E_{glob}$), small-worldness (σ) and assortativity (*a*). Such topological properties are strongly influenced by more fundamental features of the network, including the number of nodes, number of connections, and degree distribution. To control these effects, network measures for the empirical networks were compared with those for 1000 randomized networks generated using a random rewiring process (Maslov and Sneppen, 2002). At a nodal level, we estimated the degree centrality as the number of edges connecting each node to the rest of the network and the participation coefficient as a measure of the diversity of intermodular connections at each node. Global topological metrics were computed using the Brain Connectivity Toolbox (https://sites.google.com/site/bctnet/) (Rubinov and Sporns, 2010).

High degree nodes, also known as hubs, are often densely inter-connected to form a rich club. The rich club coefficients (Φ(r)) of a thresholded network were computed as the sum of edges within the subgraph defined by retaining only nodes with degree greater than an arbitrary threshold. The rich club metric was normalized ($r_{norm}\left(k\right)$) by computing the ratio of the rich club coefficient of the real network (Φ(k)) to the mean rich club coefficient of 1000 randomized networks ($\Phi_{rand}\left(k\right)$). Each network was decomposed into a set of modules, each module comprising a community of nodes that were densely connected to each other but sparsely connected to nodes in other modules (Fig. 1C). This community partitioning was performed using the Louvain algorithm (Blondel et al., 2008) with two resolution parameters (γ = 1 and γ = 2) in conjunction with consensus clustering (Lancichinetti and Fortunato, 2012). To optimise this procedure, the Louvain algorithm, implemented in the Brain Connectivity Toolbox, maximizes the metric defined by Newman (2006) comparing the density of intra-modular connections to the density expected to occur by chance in a random network:

(1) $$Q=\frac{1}{4m}\sum_{ij}\left(A_{ij}-\frac{k_{i}k_{j}}{2m}\right)\delta \left(s_{i},s_{j}\right)$$

where *m* is the total number of edges in a network, $A_{ij}$ is the adjacency matrix, $k_{i}$ and $k_{j}$ are the degree of nodes *i* and *j*, $\delta \left(s_{i},s_{j}\right)$ is the Kronecker delta, and $s_{i}$ and $s_{j}$ are the communities of nodes *i* and *j*, respectively. Because the Louvain algorithm is non-deterministic, we used consensus clustering to detect a stable consensus community structure. This modular decomposition algorithm was applied 1000 times. A (152 × 152) consensus matrix (M) was created defining each element $m_{ij}$ as the number of times that node *i* and node *j* had been assigned to the same module. Finally, the community partitioning algorithm was applied to the consensus matrix, providing a stable modular structure of the original network (Kwak et al., 2009).

### Gene co-expression within modules derived from SCN and von Economo

To assess the spatial overlap between SCN communities and gene co-expression, we averaged the gene co-expression values between each pair of regions located within the same SCN module. As this intra-modular gene co-expression is affected by trivial characteristics of the community partition, raw values were compared with those derived from random modular partitions. To test against appropriately designed surrogate data, 1000 pseudo-random communities were created by iteratively permuting the module labels associated with pairs of nodes located at the same distance from each module's centroid. This procedure randomly shifts the position of the modules along the cortex without splitting apart the components of the module (Figure S2) (Bethlehem et al., 2017). The resulting null distribution of community partitions preserves the number and size of modules, as well as the spatial contiguity of the empirical community partition. The 95th percentile of the null distribution was used as a statistical threshold to retain or reject the null hypothesis of no significant gene co-expression within modules. Fig. 1D illustrates the estimation of the mean gene co-expression within the two community partitions considered in this paper: modules derived from the SCN, and the von Economo atlas (Figure S3). This atlas groups brain regions according to cytoarchitectonic criteria (von Economo, 1929). Thus, the complete cortex was divided into: (i) granular cortex; primary motor/precentral gyrus, (ii) granular association isocortex Type I, (iii) granular association isocortex Type II, (iv) secondary sensory cortex, (v) primary sensory cortex. Due to their unique cytoarchitectonical features (vi) limbic regions (including entorhinal, retrosplenial, presubicular and cingulate) and (vii) insular cortex (which contains granular, agranular and dysgranular regions) were considered as separate classes (Seidlitz et al., 2017, Vasa et al., 2017, Vértes et al., 2016).

## Results

### Topological and spatial properties of the transcriptional brain network (TBN) and the structural covariance network (SCN)

**Fig. 2:**
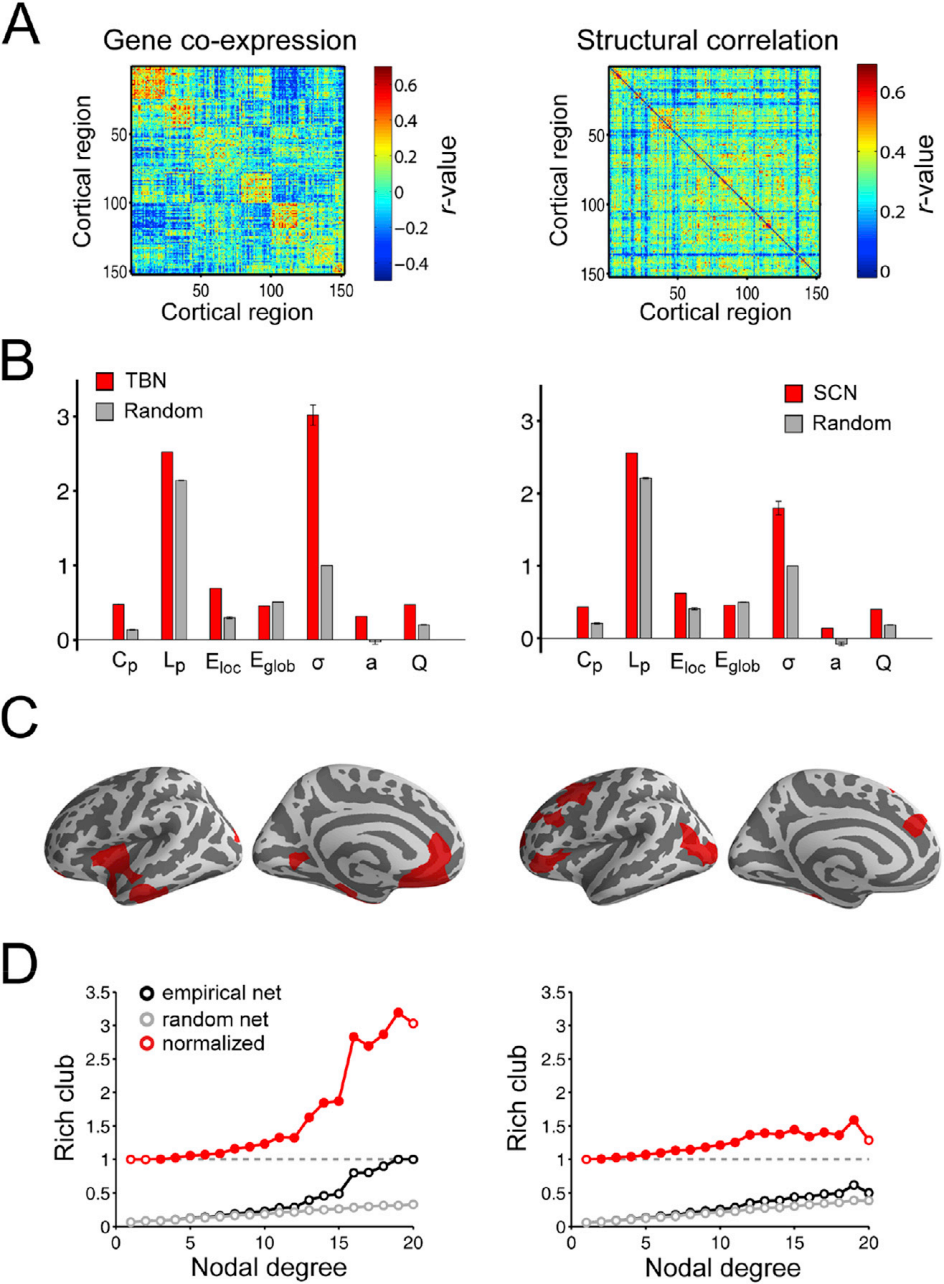
**Global topology of gene co-expression (left) and structural covariance networks (right). A**: Gene co-expression matrix and structural covariance matrix identically ordered in alignment with the modular community structure of the transcriptional network. **B:** Global topological metrics estimated in TBN, SCN and comparable random networks: C_p_ = clustering coefficient; L_p_ = path length; E_loc_ = local efficiency; E_glob_ = global efficiency; σ = small-world; a = assortativity; Q = modularity. Error bars represent standard deviations. **C:** Top 10% of nodes with highest degree (hub nodes) **D**: Rich club coefficient curves for TBN, SCN and random networks.

Following thresholding to 10% edge density, nodes in both the TBN and SCN were part of a single connected component. Topologically, both networks were small-world (as defined by greater than random clustering combined with near random path length or global efficiency) with a modular community structure and a rich club of highly interconnected hub nodes located in different areas (Fig. 2). These properties were also observed when graphs with 5% and 15% connection density were considered (Figure S4 and S5).

**Fig. 3:**
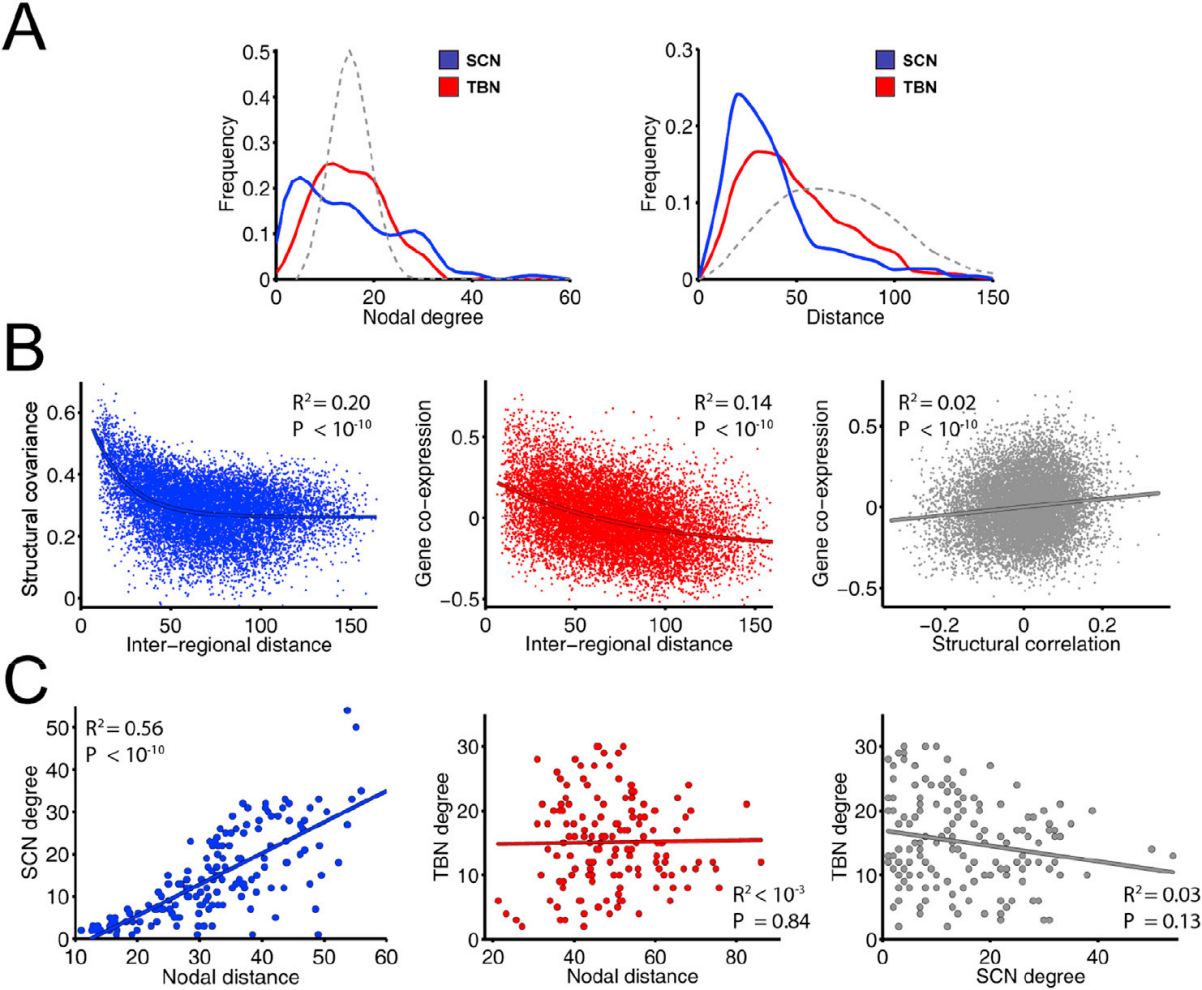
**Nodal topology and connection distance of structural covariance network (SCN) and transcriptional brain network (TBN). A.** (*left*) Degree distribution of both networks (solid lines) and a comparable random network (dashed line); (*right*) connection distance distribution of both networks (solid lines) and a comparable random network (dashed line). B. (*left*) Effect of inter-regional distance on structural covariance; *(middle)* effect of inter-regional distance on gene co-expression; and (*right*) gene co-expression versus structural covariance. C. (*left*) Degree versus connection distance in the SCN; (*middle*) degree versus connection distance in the TBN; and (*right*) nodal degree in TBN versus nodal degree in SCN.

The degree distribution of both networks was fat-tailed compared to a random graph (Fig. 3A, left panel), the SCN having more highly-connected hubs than the TBN. In both networks, the distance distribution was skewed towards shorter distances, compared to a random graph, but there was a tail of long distance connections that was particularly prominent in the TBN (Fig. 3A, right panel). Structural covariation strength and gene co-expression strength both decreased monotonically as a function of increasing physical distance between nodes (R^2^ = 0.20 and R^2^ = 0.14, respectively; Fig. 3B, first and second panel). Akaike's information criterion (AIC) demonstrated that an exponential function of distance provided a better fit than a linear function for both structural covariance ($AIC_{linear}=-0.33$ and $AIC_{exponential}=-0.55$) and gene co-expression ($AIC_{linear}=5.13$ and $AIC_{exponential}=5.11$). Gene co-expression and structural covariance were significantly correlated (R^2^ = 0.05, P < 10^−10^; Figure S6). This relationship remained significant after correcting for inter-regional distance (R^2^ = 0.02, P < 10^−10^; Fig. 3B, third panel). Nodal degree was significantly related to the average Euclidean distance between connected nodes in the SCN (R^2^ = 0.56, P < 10^−10^; Fig. 3C, first panel), i.e., high degree hubs had more long-distance connections to other nodes; but there was no relationship between distance and degree in the TBN (R^2^ < 10^−3^, P = .84; Fig. 3C, second panel). Nodal degree was not correlated between the structural covariance and transcriptional networks (R^2^ = 0.03, P = .13; Fig. 3C, third panel), i.e., the high degree hubs of the SCN did not correspond to the hubs of the TBN.

### Whole genome co-expression in relation to the modular community structure of the SCN

**Fig. 4:**
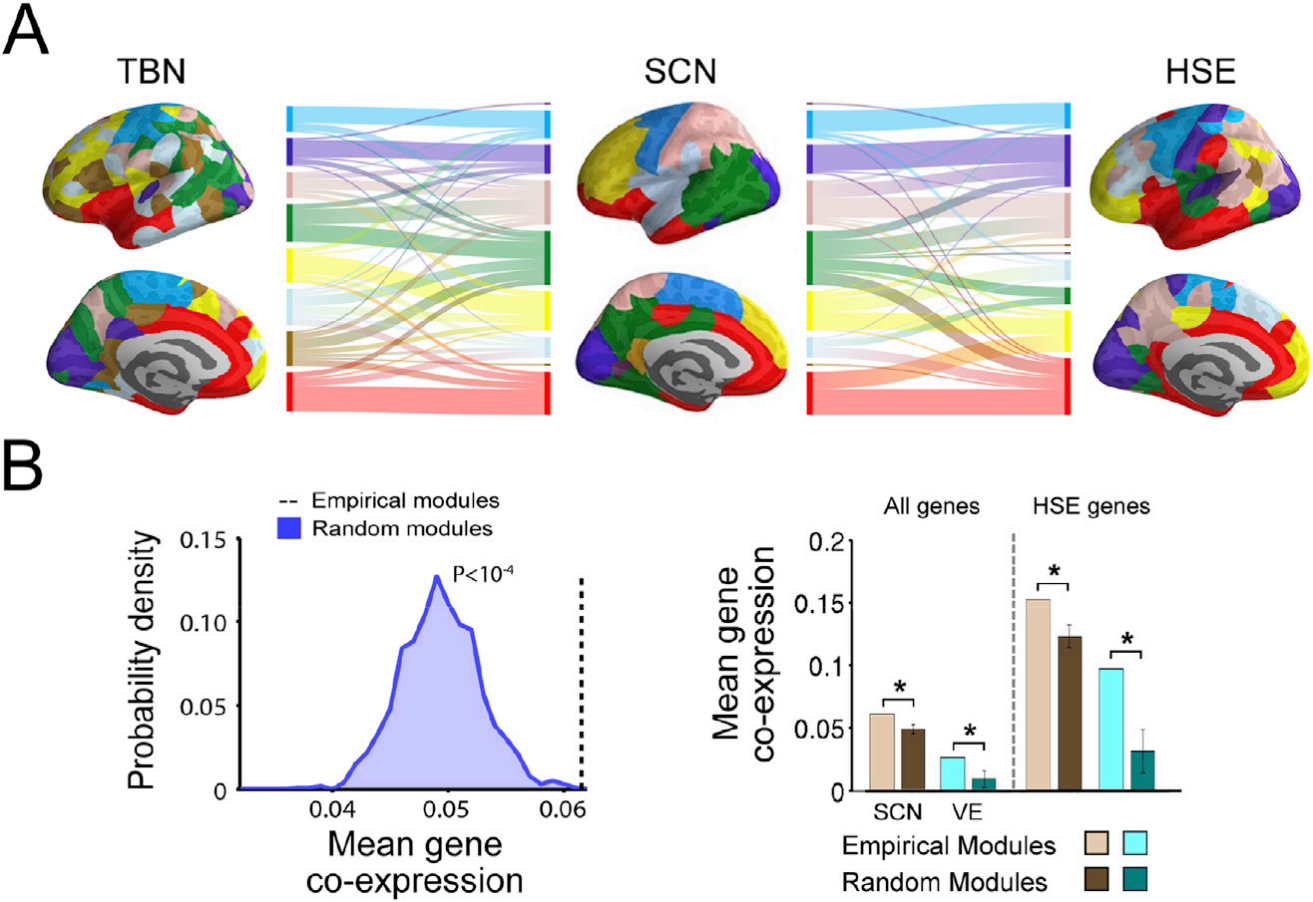
**Community structure of the structural covariance network and transcriptomic brain network. A:** Modular decomposition of the whole genome transcriptional brain network (TBN; 7 modules; left); modular decomposition of the structural covariance network (SCN; 9 modules; middle); modular decomposition of the transcriptional brain network restricted to HSE genes (8 modules, right); and two alluvial diagrams showing how regions were aligned to the same or different modules in the SCN and the two transcriptional networks;. **B**: (*left*) Distribution of whole genome co-expression between regions assigned to the same random modules compared to the whole genome co-expression between regions assigned to the same modules of the empirical SCN; (*right*) co-expression of the whole genome and the HSE gene set within each module of the SCN, within each cytoarchitectonic class of the von Economo (VE) atlas, and within comparable random modules. Error bars represent standard deviations.

The spatial location of TBN modules was partially overlapping with the community structure of the structural covariance network (Fig. 4A). Gene co-expression was significantly higher between regional nodes that belonged to the same module of the SCN than between nodes that belonged to randomly defined modules that preserved the number, size and spatial contiguity of the SCN modules (Fig. 4B, left panel; P < .001). This result was replicated for different SCN connection densities (5%, 10% and 15%) and modularity resolution parameters (γ = 1 and γ = 2) (all P < .05; Figure S7). Gene co-expression was also significantly increased within regions grouped according to the cytoarchitectonic criteria of von Economo (P < .02; Fig. 4B, right panel; (von Economo, 1929)). In other words, gene co-expression was increased between regions that belonged to the same cytoarchitectonic class.

### HSE gene expression and structural covariation

**Fig. 5:**
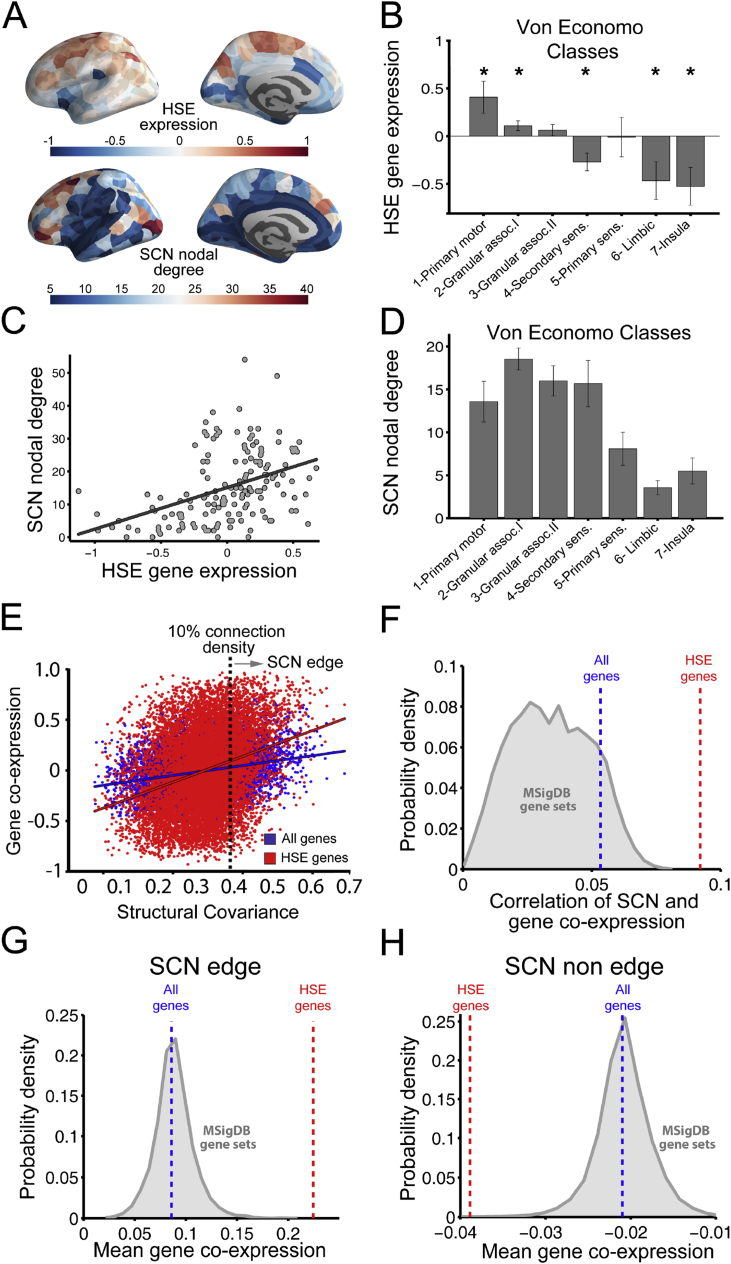
**Structural covariance and HSE gene co-expression**. **A:** HSE gene expression (top) and SCN nodal degree (bottom). **B:** HSE gene expression in different cytoarchitectonic classes defined by the von Economo atlas. **C:** Nodal degree in structural covariance network versus HSE gene expression. **D:** Mean nodal degree in different cytoarchitectonic classes defined by the von Economo atlas. **E:** Co-expression (whole genome, blue; or HSE genes only, red) versus structural covariance. Dashed vertical line indicates the threshold value of structural covariance used to define a binary graph of edges and non-edges. **F:** Correlations between structural covariance and (i) whole genome co-expression (scalar value represented as a dashed blue vertical line), (ii) HSE gene co-expression (scalar value represented as a dashed red vertical line), (iii) co-expression of each of the 5917 gene ontologies defined in the MSigDB collection (represented in grey as a probability density). Each value of the distribution represents the proportion of MSigDB gene sets showing the corresponding correlation with SCN. **G:** Co-expression (whole genome, blue; HSE genes only, red; and each of the 5917 gene ontologies, grey distribution) for SCN edges. **H:** Co-expression (whole genome, blue; HSE genes only, red and each of the 5917 gene ontologies, grey distribution) for SCN non edges (unconnected regions).

Expression of the HSE gene set was explored due to its particular transcriptional profile in supragranular layers of the human cortex and its association with long-range cortico-cortical connectivity (Zeng et al., 2012). HSE genes showed a heterogeneous expression and co-expression pattern across the cortex that partially overlapped with the anatomical patterning of modular structure (Fig. 4A) and nodal degree (Fig. 5A) of the SCN. HSE genes were over-expressed (compared with the whole brain mean expression level) in von Economo classes 1 (granular, primary motor cortex) and 2 (association cortex); whereas HSE genes were under-expressed in classes 4 (secondary sensory cortex), 6 (limbic regions) and 7 (insular cortex) (Fig. 5B; P < .005, FDR-corrected). HSE gene expression was significantly positively correlated with nodal degree (R^*2*^ = 0.17; P < 10^−7^; Fig. 5C) and participation coefficient (R^*2*^ = 0.23; P < 10^−8^; Figure S8) of the SCN. SCN nodal degree was not correlated with community size (Figure S9) and it was higher in von Economo classes 1,2,3 and 4 than in classes 5,6 and 7 (Fig. 5D).

Correlation between structural covariance and gene co-expression was higher for HSE genes (R^*2*^ = 0.09, P < 10^−10^) than when the complete genome was considered (R^*2*^ = 0.053, P < 10^−10^; permutation test) and this difference was statistically significant (*F*_1,11475_ = 485, P < 10^−10^, after regressing out the effect of the distance; Fig. 5E). Additionally, correlation between structural covariance and gene co-expression was higher for the HSE genes than for all the other gene ontologies defined in the MSigDB collection (Fig. 5F). After the HSE genes, cation channel complex and multicellular organismal signaling gene ontologies showed the strongest correlation with structural covariance of the 5917 ontologies analyzed (R^*2*^ = 0.078 and R^*2*^ = 0.077, respectively).

Gene co-expression between connected regions in the structural covariance network (SCN edges) was higher for HSE genes than for the complete genome and for all other 5917 gene ontologies (Fig. 5G). By contrast, pairs of regions that were unconnected in the structural covariance network at 10% connection density, i.e., SCN non-edges, showed gene co-expression levels close to zero that are more negative for HSE genes than for the whole genome and for the gene ontologies co-expression, suggesting that regions with dissimilar CT pattern (SCN non-edge) have (weakly) opposite HSE expression levels (Fig. 5H). These results were also replicated at connection densities of 5% and 15% (Figure S10 and S11).

### Effects of spatial distance on structural covariance and gene co-expression

A linear function of inter-regional log distance explained 16% of the variance in strength of structural covariance (Figure S12). HSE gene co-expression explained 9% of the variance in strength of structural covariance; and cytoarchitectonic class membership explained 6% of the variance. Linear multiple regression demonstrated that the combination of log distance and HSE gene co-expression collectively explained 22% of the variance in strength of structural covariance.

## Discussion

Here we have compared the organization of a structural covariance network, derived from cortical thickness measurements in 152 left-hemisphere regions in 296 healthy young participants, to the organization of a transcriptional network, derived from gene expression measurements in the same 152 regions in 6 adult post mortem brains. Since transcriptionally and histologically similar brain regions are more likely to be anatomically connected (Goulas et al., 2016), and since structural covariance is a putative marker of anatomical connectivity, we made two, hypothetically related predictions: i) that co-expression of genes should be correlated with the strength of structural covariance between regions; and ii) that the topology of the structural covariance network should be coupled to the topology of the transcriptional brain network. Considering co-expression of the nearly complete genome, we found no more than modest support for these predictions: the two networks had qualitatively similar topology, their edge weights were significantly correlated and there was evidence for greater whole genome co-expression between brain regions that were assigned to the same topological module of the SCN's community structure. But there was stronger evidence in support of links between structural covariance or SCN topology and regional co-expression of a much smaller set of (19 HSE) genes, known to be enriched specifically in human supra-granular cortex.

### Gene expression and co-expression in the brain

The neocortex displays a remarkable conservation of gene expression across individuals (Hawrylycz et al., 2015), cortical areas (Hawrylycz et al., 2012) and species (Oldham et al., 2006, Zeng et al., 2012). In accordance with the cortical homogeneity of the transcriptional profile, we found that most of the inter-regional gene co-expression values were extremely significant. Nevertheless, regional differences in cortical gene transcription are subtle but important. Studies based on non-human animals reveal that different cell types display a robust transcriptional signature where neurons, oligodendrocytes, astrocytes and microglia tend to up-regulate and down-regulate the expression of specific subsets of genes (Grange et al., 2014, Ko et al., 2013, Lein et al., 2007). This transcriptional signature of cell type-specific genes can be used to define both major compartments and smaller anatomical structures of the mouse brain (Grange et al., 2014, Ko et al., 2013). In humans, the grouping of genes according to their co-expression pattern also reveals a modular structure that distinguishes the major cell classes of the underlying brain tissue (Eising et al., 2016, Oldham et al., 2009). Prior studies of the Allen Human Brain Atlas showed, in agreement with mouse studies, that the gene expression pattern reflects the specialization of primary sensorimotor areas and subdivisions of the frontal lobe (Hawrylycz et al., 2012). In keeping with these results, we found that gene co-expression was significantly related to spatial proximity: two cortical regions that are close together will have more similar gene expression profiles (Bernard et al., 2012, Hawrylycz et al., 2015, Hawrylycz et al., 2012, Lein et al., 2007). It has been proposed that the effect of anatomical closeness on transcriptional similarity reflects phylogenetic and ontogenetic distance between regions (Zapala et al., 2005).

### AIBS samples matching

The method for matching AIBS samples to MRI regions of interest in the present study does not rely on manual interventions and relies on more conservative assumptions about homogeneity of gene expression across short distances in the brain than previous studies. In particular, prior work assigned each MRI region of interest to the anatomical structure (as defined in the AIBS dataset) containing the nearest AIBS sample across all donors and then averaged gene expression from all samples falling within that AIBS anatomical region (Vértes et al., 2016, Whitaker et al., 2016). In contrast, French and Paus (2015) took a more straightforward approach where a sample was mapped into a FreeSurfer region if its MNI-152 coordinates were inside a FreeSurfer cortical region. However, this required visual inspection due to spatial distortions being introduced by coregistration of donors' brains to standard MNI-152 space. In this work, we provide a fully automated method for matching MRI parcellation with Allen Brain Atlas samples in each donor's native space in order to take into account inter-individual difference of cortical morphology.

### Transcriptional brain networks

Gene co-expression networks traditionally refer to networks where the nodes represent genes and edges denote significant associations of expression levels across samples in a dataset (for a review, see Fontenot and Konopka, 2014). In this type of network, edges link pairs of genes that are over/under expressed in the same tissue. In contrast, the transcriptional brain network, proposed here, describes transcriptional relationships (edges) between spatially delimited cortical regions (nodes). Previous studies have shown a strong association between resting-state fMRI connectivity and transcriptional similarity between brain regions (Hawrylycz et al., 2015, Richiardi et al., 2015). In this study, we used, for the first time, network analysis and graph theoretical measures to explore the topological characteristics of the TBN in order to compare the transcriptional relationships between brain regions to the human brain connectome based on anatomical measures derived from MRI data.

### Whole genome co-expression and structural covariance

Using graph theoretical methods to analyse the topology of the transcriptomic brain network we found evidence for topological segregation (high clustering coefficient and modules) and topological integration (short path length, high global efficiency and a rich club). This was a qualitatively similar profile to the complex topology of the structural covariance network. However, the extent of overlap between whole genome co-expression and structural covariance networks was limited. For example, there was a significant but weak correlation between edge-wise whole genome co-expression and structural covariance; the strength of structural covariance decayed much faster as a function of physical distance than the strength of whole genome co-expression; and only in the SCN (not TBN) was nodal degree significantly related to mean connection distance. The coupling between whole genome co-expression and structural covariance strengthened somewhat when the analysis was restricted to the 10% of network edges representing the strongest correlations in cortical thickness between regions (Fig. 5). Analysis of the modular community structure of both networks provided stronger (but still modest) evidence of overlap between the structural covariance network and the whole genome transcriptome. Whole genome co-expression was significantly stronger between pairs of nodes that belonged to the same module of the experimentally estimated SCN compared to pairs of nodes belonging to a null distribution of pseudo-modules, generated by permuting empirical modules on the cortical surface while preserving module size, contiguity and distance.

These results should be considered in the context of some relevant prior studies (Chen et al., 2011, Chen et al., 2013, Chen et al., 2012) that evaluated genetic influences on cortical areal expansion by correlating the individual genetic profile with the deformation (stretching or compression) needed to map each subjects's cortical surface into a standard space. The association between genotype and area expansion was used to define the boundaries of a genetic subdivision of the cortex and depict its genetic patterning. Resulting genetic subdivision revealed a hierarchical, modular organization consistent with specialized functional and lobar subdivisions. Moreover, this genetic subdivision followed closely the developmental changes of cortical thickness, with age-related cortical shrinkage trajectories varying as a function of inter-regional genetic similarity (Fjell et al., 2015). Genetic associations between regional measures of cortical thickness have been previously described by Docherty et al. (2015). This twin-based study revealed that genetic relationships between structurally-determined regions exhibit small-world properties (Docherty et al., 2015). Using the AIBS dataset (Wong et al., 2017) have recently described that cortical thinning in adolescence is associated with the expression of genes coding for glucocorticoid and androgen receptors. Fulcher and Fornito (2016) showed that transcriptomic correlations are stronger between hub nodes than between non-hubs of the mouse brain connectome derived from tract-tracing data.

### HSE gene co-expression and structural covariance

For the HSE gene set, we concentrated on the relationships between HSE gene co-expression and structural covariance. HSE gene co-expression was significantly correlated with structural covariance; high degree hubs in the structural covariance network had higher levels of HSE gene expression than less central, low degree nodes; and nodes that belonged to the same SCN module or the same cytoarchitectonic class had higher levels of HSE gene co-expression than pairs of regions that belonged to different modules or classes.

These results constitute further evidence that structural covariance and network topology is linked to gene co-expression and are compatible with what is known about HSE genes. HSE genes are overexpressed in the supragranular layers (II and III) of the cerebral cortex in humans, but under-expressed in the same layers of mouse cortex (Zeng et al., 2012). This small but substantial species-differential expression is hypothesized to drive the shift from predominantly cortico-subcortical connectivity in non-primate mammals to the major emphasis on cortico-cortical projections in the human brain (Zeng et al., 2012). This is consistent with the overexpression of HSE genes in von Economo classes 1 (precentral gyrus) and 2 (association cortex) described here because, according to histological findings, these regions have an especially prominent supragranular layer III that includes pyramidal neurons as well as intra-cortical axons (von Economo, 1929). We hypothesize that the high levels of SCN nodal degree in these regions may be related to the large proportion of the full thickness of the cortex that is taken up by layer III in these areas, as well as its role as a principal source of cortico-cortical efferent projections. Similarly, neuronal size in layer III is the only cytoarchitectonic metric that has been previously associated with anatomical connectivity strength derived from DTI (van den Heuvel et al., 2015). Two prior fMRI studies in humans have shown that HSE gene (co)-expression is related to functional connectivity between cortical areas. Krienen et al. (2016) reported stronger HSE transcriptional similarity within than between resting state fMRI networks (RSNs), which share many similarities to modules. Likewise, Vértes et al. (2016) showed that HSE genes are significantly over-represented among the genes that are most over-expressed in cortical areas with high inter-modular degree and long mean connection distance. The data reported here add to this literature by demonstrating for the first time that HSE gene co-expression is also linked to MRI measures of structural covariance and brain anatomical network topology. Thus the evidence is growing that HSE genes play an important role in the formation of human connectome topology, especially high degree hubs and long distance connections. It would be interesting in future studies to explore the effects of sequence variation in HSE genes on human connectome phenotypes and their development.

### Methodological issues

First, it is important to note that the gene expression dataset provided by AIBS included six left hemispheres but only two right hemispheres, due to the expression similarities between hemispheres (Hawrylycz et al., 2012). Given the severely reduced sampling of the right hemisphere, we pooled all the samples into the left hemisphere and, consequently, only MRI data from the left hemisphere was included in the analyses. As CT is highly similar between homologous regions across hemispheres (R^*2*^ = 0.83, P < 10^−59^; Figure S13), our results may be generalized to whole brain or inter-hemispheric connectivity; however, additional gene expression data in the right hemisphere will be necessary to confirm this hypothesis. Third, the tissue samples used in the microarrays were not homogenously distributed across the cortex. As a result, gene expression of each cortical region was calculated by averaging a different number of AIBS samples, leading to a variable signal-to-noise ratio across regions. Fourth, AIBS data were based on 5 male donors and one female, with a mean age of 42.5 years, whereas the MRI data were collected from 296 healthy gender-balanced subjects with a mean age of 19.1 years. Although structural covariance remains similar after age-correction of cortical thickness (R^*2*^ = 0.96, P < 10^−100^; Figure S14), age- and gender-related changes in brain gene expression (Berchtold et al., 2008), as well as other individual differences, may be an important confounding factor when comparing transcriptomic and neuroimaging data.

## Conclusions

Structural covariance based on MRI measurements of cortical thickness, and a transcriptional brain network had similar complex topological properties, showing organizational patterns that partially, but not completely, overlapped. The high degree hubs of structural covariance networks were coupled specifically to regional expression and co-expression of a set of genes known to be important for long-range cortico-cortical connectivity of the human brain.

## Conflicts of interest

ETB is employed half-time by the University of Cambridge and half-time by GlaxoSmithKline (GSK); he holds stock in GSK.

## Funding

This work was supported by a strategic award from the Wellcome Trust to the University of Cambridge and University College London (095844/Z/11/Z): the Neuroscience in Psychiatry Network (NSPN). Additional support was provided by the NIHR Cambridge Biomedical Research Centre. KJW is supported by a Mozilla Science Lab Fellowship and the Alan Turing Institute under the EPSRC grant EP/N510129/1. PEV is supported by a Medical Research Council Bioinformatics Research Fellowship (Grant No. MR/K020706/1). FV was supported by the Gates Cambridge Trust. MS was supported by the Winston Churchill Foundation of the United States.

## Appendix A Supplementary data

Romero-Garcia_TBN_SM Romero-Garcia_TBN_SM
