# Supplementary material for: Structural covariance networks are coupled to expression of genes enriched in supragranular layers of the human cortex
Source: Neuroimage. 2018 May 1;171:256–67. doi: 10.1016/j.neuroimage.2017.12.060 (PMC5883331; doi:10.1016/j.neuroimage.2017.12.060)
Supplement: Romero-Garcia_TBN_SM [file mmc1.docx]

**Supplementary Information**

**Structural covariance networks are coupled to expression of genes enriched in supragranular layers of the human cortex**

Rafael Romero-Garcia^1,§^, Kirstie J Whitaker^1,2^, František Váša^1^, Jakob Seidlitz^1^, Maxwell Shinn^1^, Peter Fonagy^3^, Raymond J Dolan^4,5,^ Peter B Jones^1,6^, Ian M Goodyer^1,6^, the NSPN Consortium^‡^, Edward T Bullmore ^1,6,7^, Petra E Vértes^1^.

^1^Department of Psychiatry, University of Cambridge, Cambridge, CB2 0SZ, UK.

^2^The Alan Turing Institute for Data Science, British Library, 96 Euston Road, London, NW1 2DB, United Kingdom

^3^Research Department of Clinical, Educational and Health Psychology, University College London, London, WC1E 6BT, UK.

^4^Wellcome Trust Centre for Neuroimaging, UCL Institute of Neurology, University College London, London, WC1N 3BG, UK.

^5^Max Planck UCL Centre for Computational Psychiatry and Ageing Research, London, WC1B 5EH, UK.

^6^Cambridgeshire and Peterborough NHS Foundation Trust, Huntingdon, PE29 3RJ, UK.

^7^ImmunoPsychiatry, Immuno-Inflammation Therapeutic Area Unit, GlaxoSmithKline R&D, Stevenage SG1 2NY, UK.

§ **Correspondence to**: Rafael Romero-Garcia PhD, University of Cambridge, Department of Psychiatry, Sir William Hardy Building, Downing Street, Cambridge CB2 3EB, UK Tel: 1223 764673, Email: rr480@cam.ac.uk

**Keywords:** Allen Brain Atlas, cortical thickness, gene expression, structural brain network, transcriptomic brain network


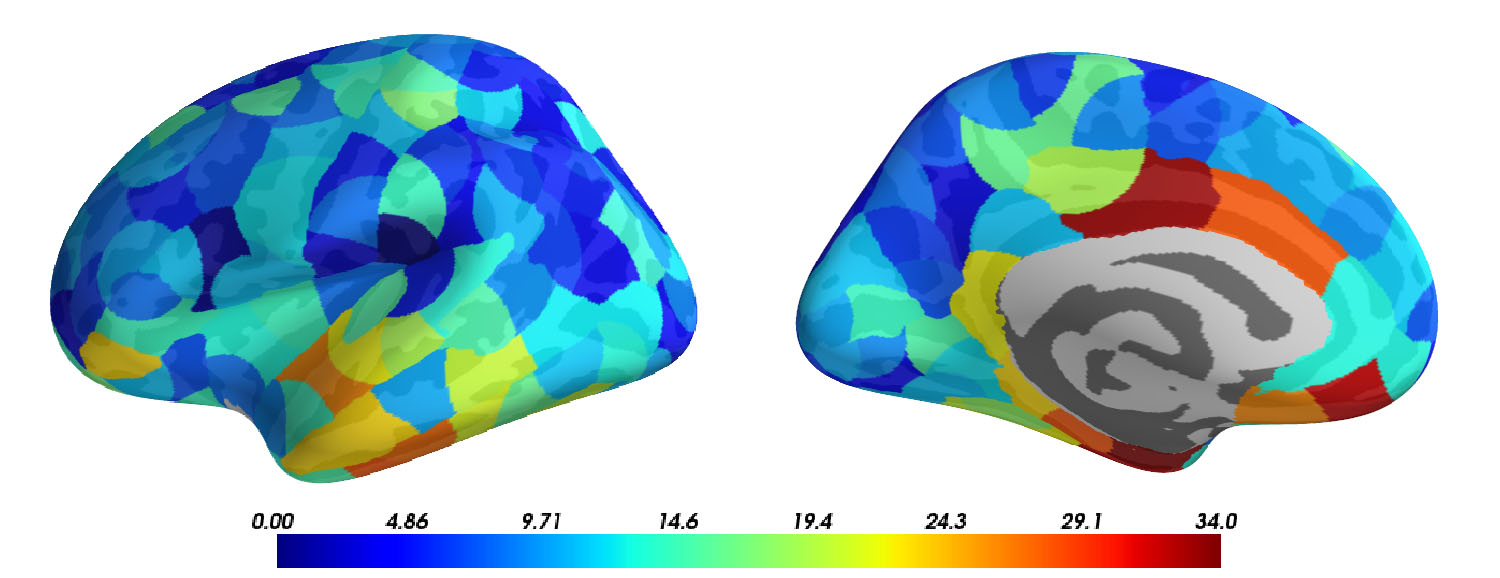


**Figure S1:** Number of AIBS samples in each cortical region. Inferior supramarginal gyrus was the only parcel that included zero AIBS samples.


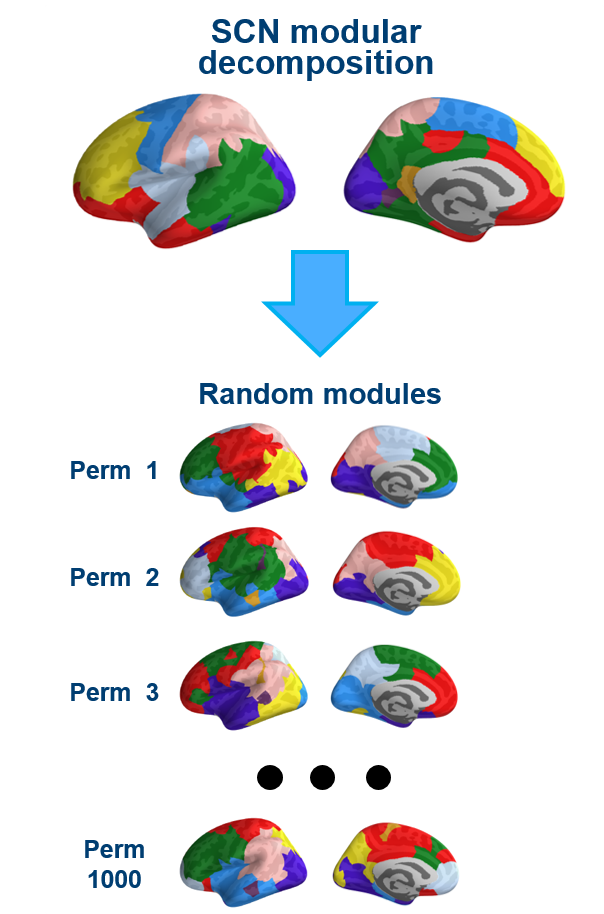


**Figure S2:** Random modular communities that preserve the number and size of modules, as well as the spatial contiguity of the empirical community partition.

**
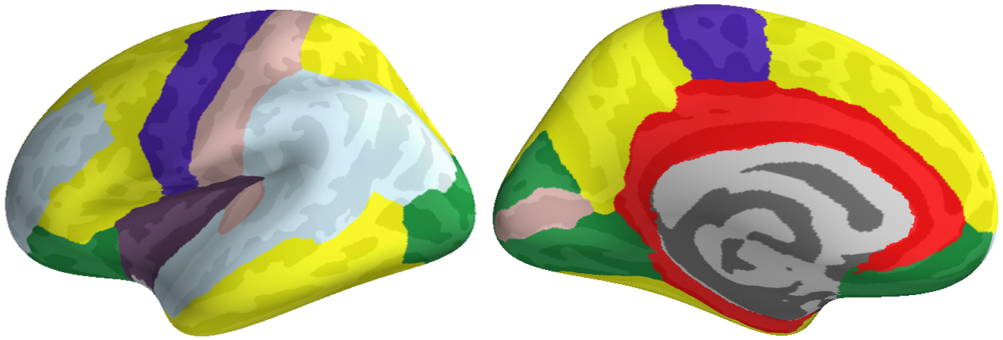
**

**Figure S3:** **Von Economo atlas.** Granular cortex; primary motor/precentral gyrus (light purple), granular association isocortex Type I (yellow), granular association isocortex Type II (grey), secondary sensory cortex (green), primary sensory cortex (pink), limbic regions (red) and insular cortex (dark purple).


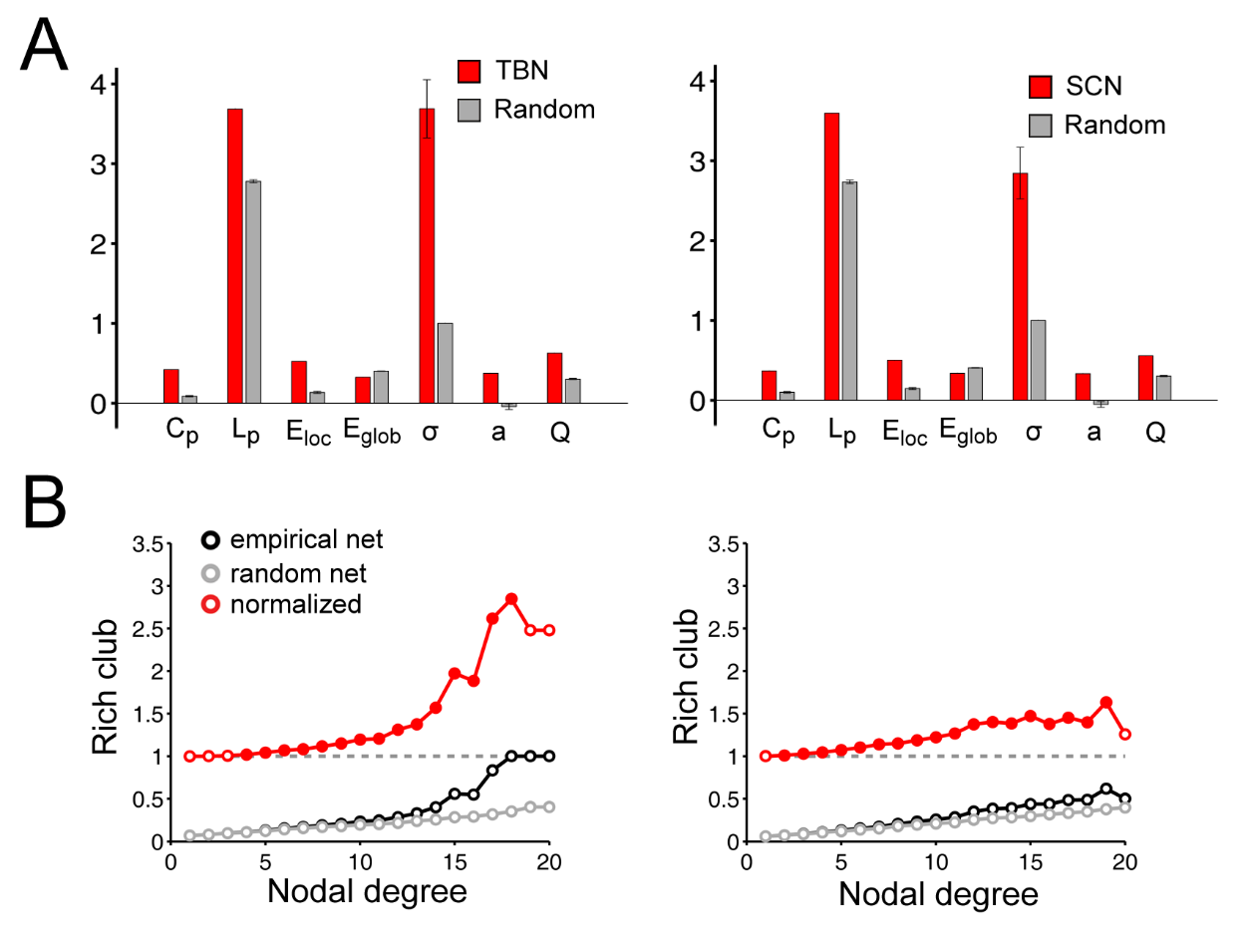


**Figure S4:** **Global topology of gene co-expression (left) and structural covariance networks (right) at 5% connection density.** **A**: Global topological metrics estimated in TBN, SCN and comparable random networks: C_p_ = clustering coefficient; L_p_ = path length; E_loc_ = local efficiency; E_glob_ = global efficiency; σ = small-world; a = assortativity; Q = modularity. Error bars represents the standard deviations. **B**: Rich club coefficient curves for TBN, SCN and random networks.


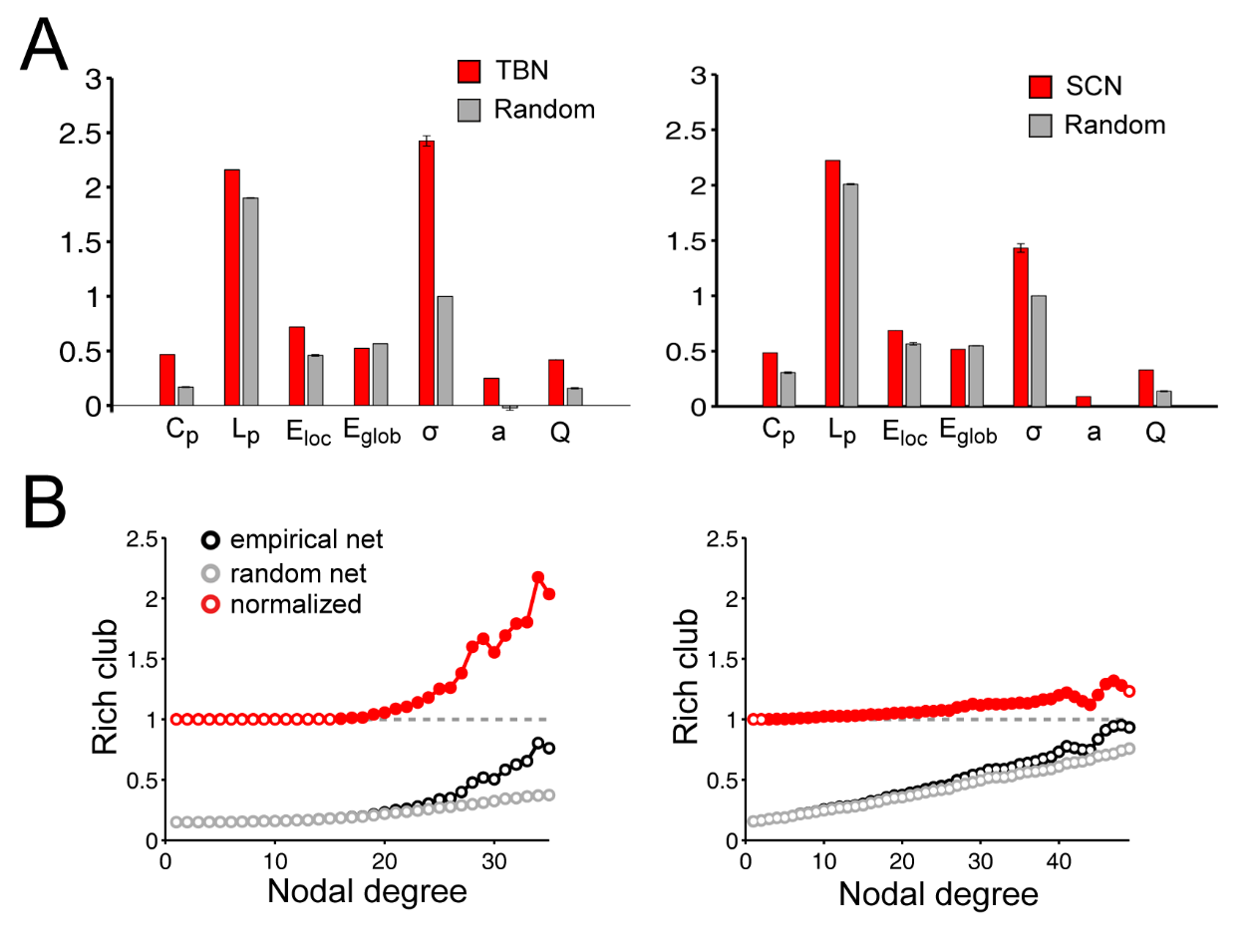


**Figure S5:** **Global topology of gene co-expression (left) and structural covariance networks (right) at 15% connection density.** **A**: Global topological metrics estimated in TBN, SCN and comparable random networks: C_p_ = clustering coefficient; L_p_ = path length; E_loc_ = local efficiency; E_glob_ = global efficiency; σ = small-world; a = assortativity; Q = modularity. Error bars represents the standard deviations. **B**: Rich club coefficient curves for TBN, SCN and random networks.


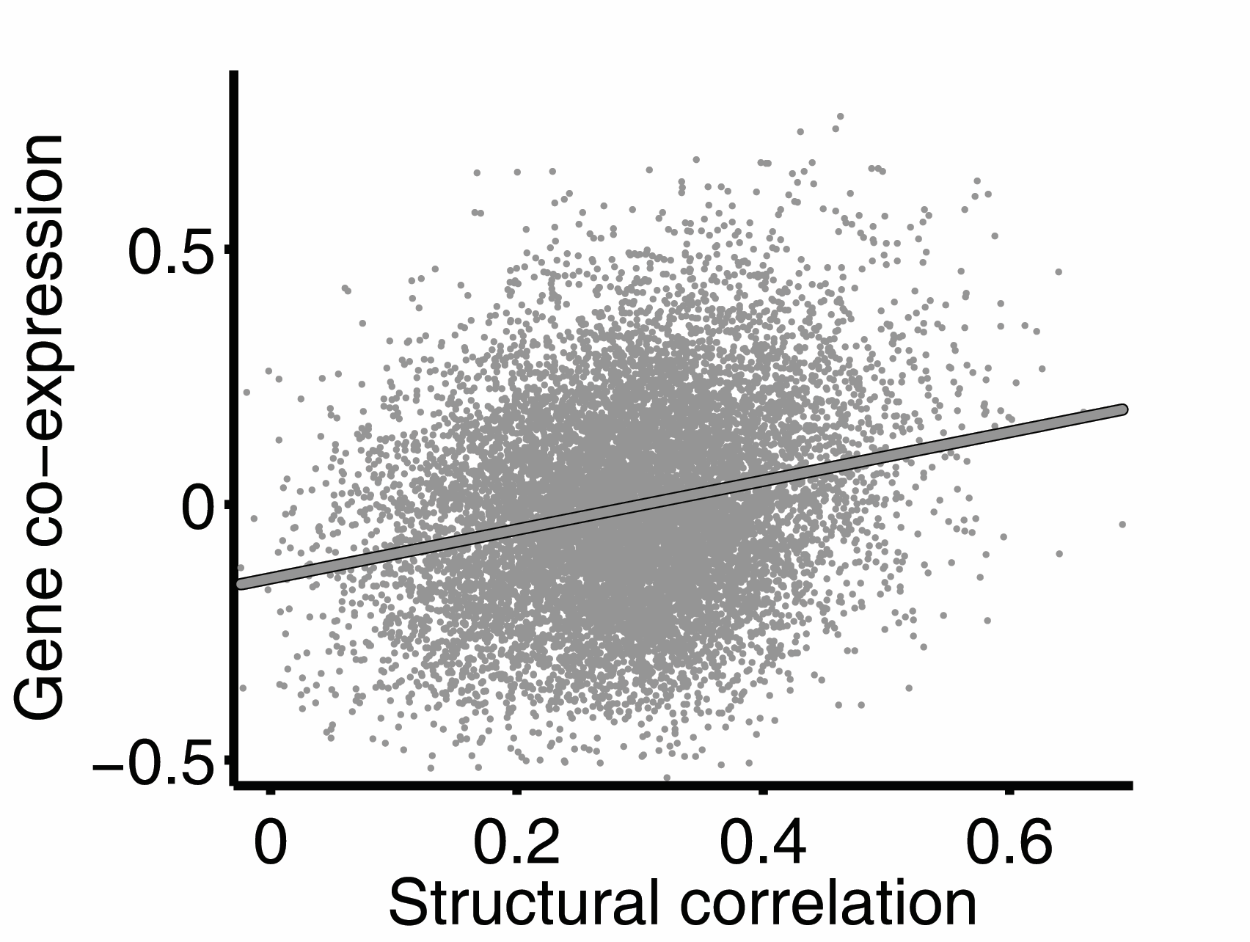


**Figure S6:** Scatterplot of gene co-expression versus structural covariance after regressing out the effect of the inter-regional distance.

**
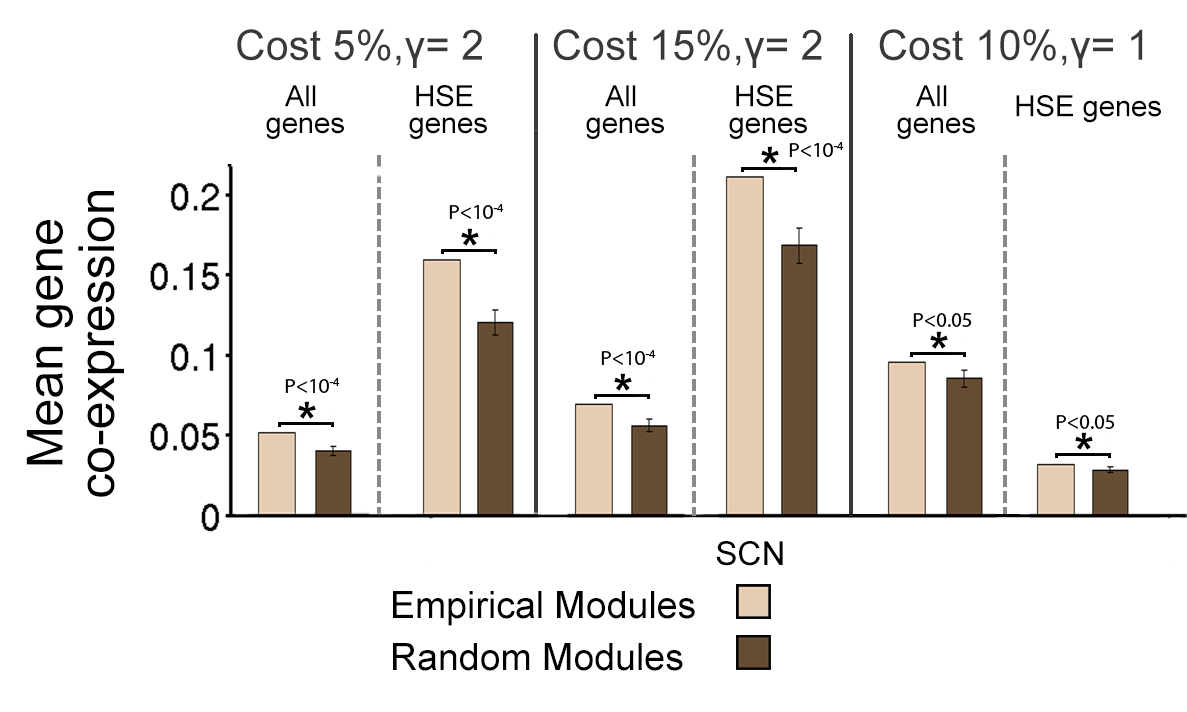
**

**Figure S7:** Mean gene co-expression between nodes located in the same SCN module for the empirical modules (light colors) and the random null modules (dark colors). Results replicated for different SCN costs (5%, 10% and 15%) and modularity resolution parameters (γ=1 and γ=2).

**
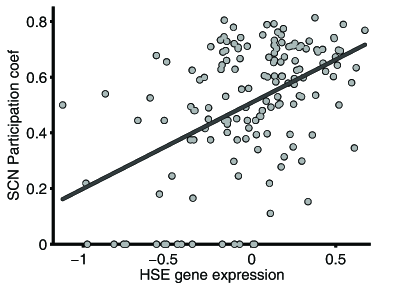
**

**Figure S8:** Association between expression of HSE genes and participation coefficient of the SCN.­

**
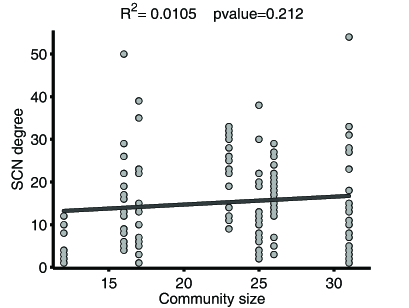
**

**Figure S9:** Nodal degree of the SCN was not associated with community size (number of regions belonging to each module).


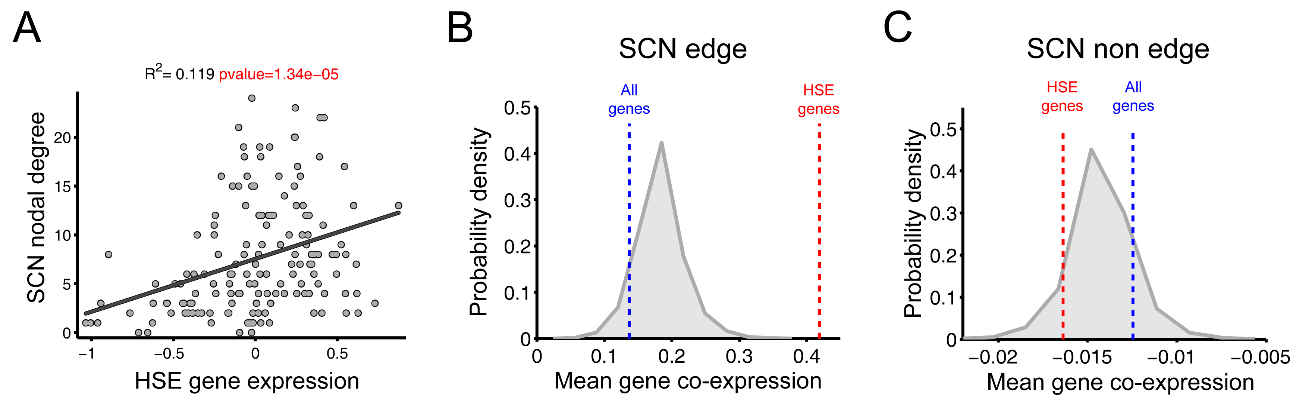


**Figure S10: Gene co-expression and SCN at 5% connection density**. **A:** Nodal degree in structural covariance network versus HSE gene expression**. B:** Co-expression (whole genome, blue; HSE genes only, red and each of the 5917 gene ontologies, grey distribution) for SCN edges. **C:** Co-expression (whole genome, blue; HSE genes only, red and each of the 5917 gene ontologies, grey distribution) for SCN non edges (unconnected regions).


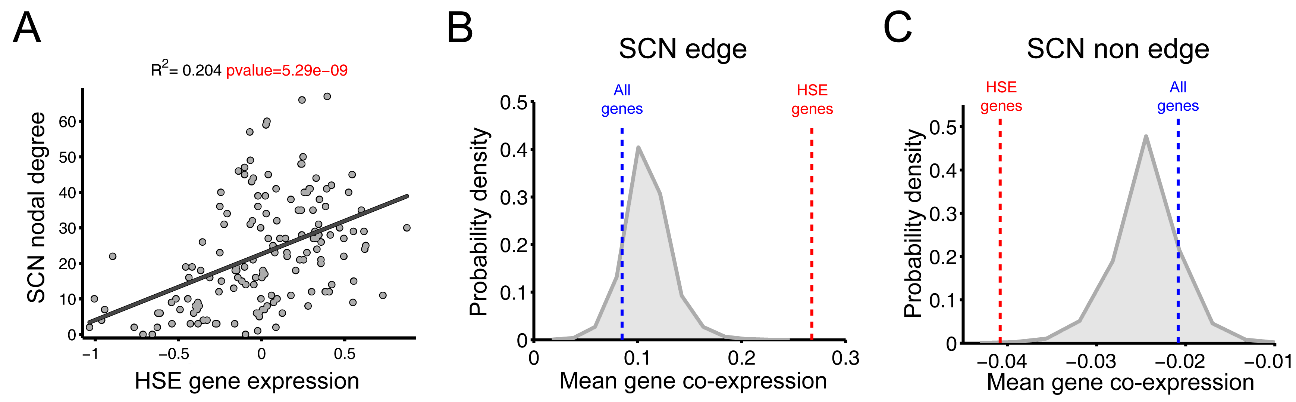


**Figure S11: Gene co-expression and SCN at 15% connection density**. **A:** Nodal degree in structural covariance network versus HSE gene expression**. B:** Co-expression (whole genome, blue; HSE genes only, red and each of the 5917 gene ontologies, grey distribution) for SCN edges. **C:** Co-expression (whole genome, blue; HSE genes only, red and each of the 5917 gene ontologies, grey distribution) for SCN non edges (unconnected regions).


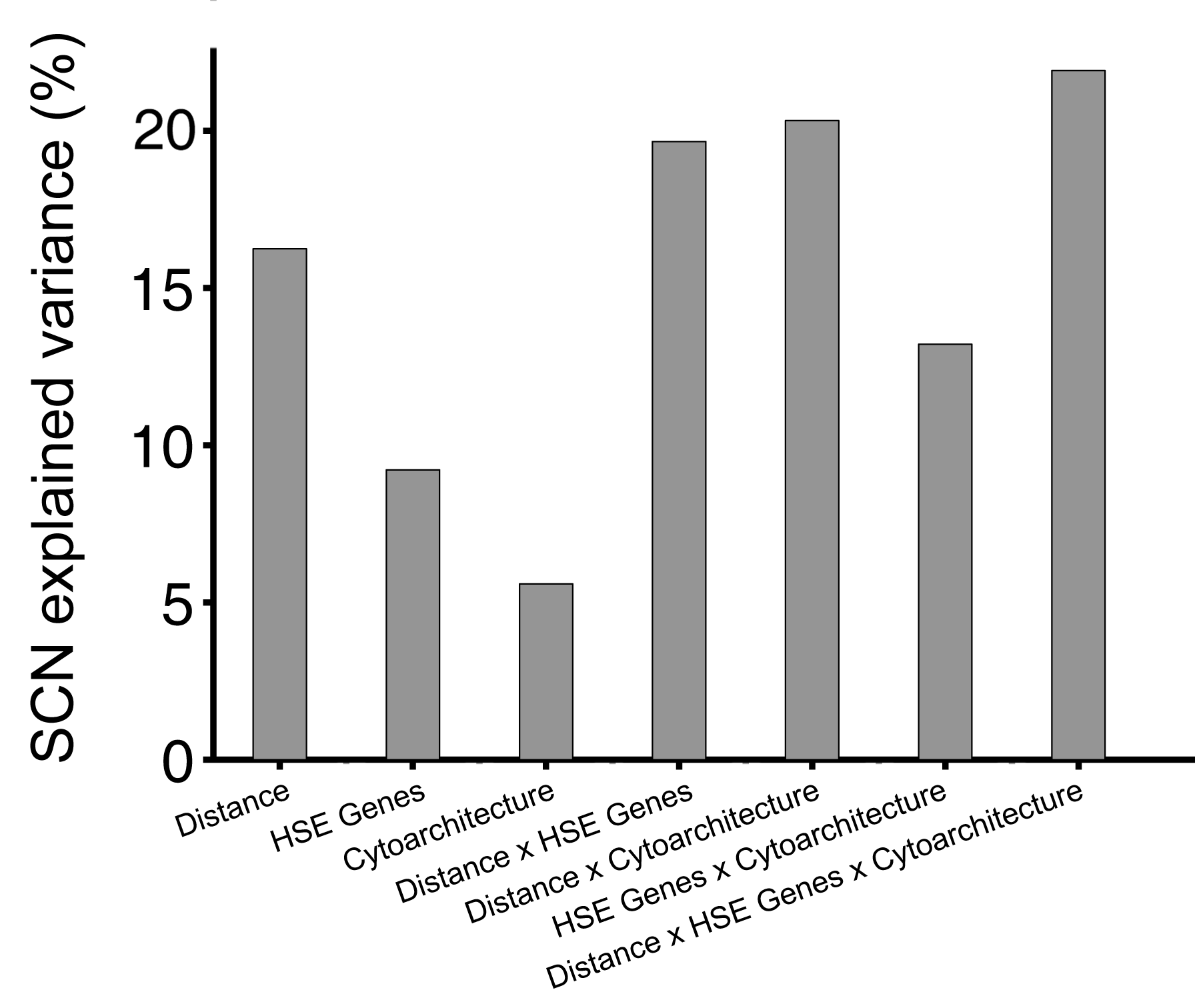


**Figure S12:** HSE gene expression has effects on structural covariation that are additive to the effects of connection distance. First column represents the SCN variance explained (R^2^) by inter-regional distance using the log value of the inter-regional distance. Second and third column show the SCN variance explained by HSE gene expression and the 7 laminar classes defined in the Von Economo atlas. The rest of the columns display the SCN variance explained by a linear multiple regression using combinations of variables.

**
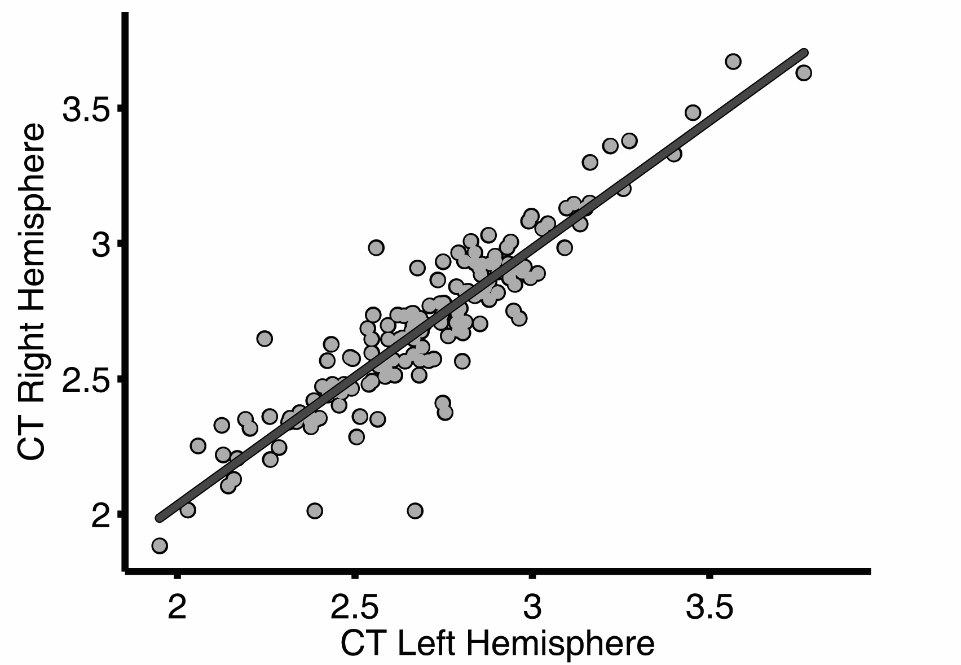
**

**Figure S13:** Association between CT values of homologous regions.


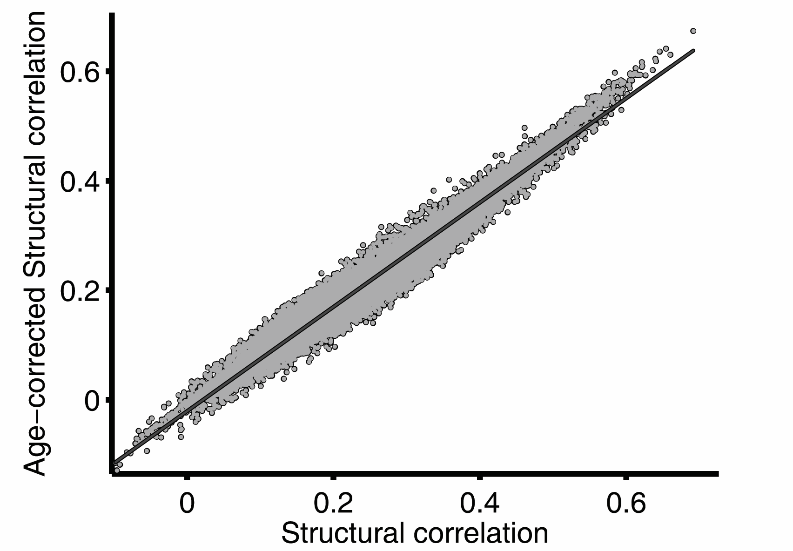


**Figure S14**. Similarity of structural correlation before and after age correction.

**NSPN Consortium member list**

**Chief investigator:**

Edward Bullmore

**Principal investigators:**

Ian Goodyer

Raymond Dolan

Peter Fonagy

Peter Jones

**Associated faculty:**

Peter Dayan

Paul Fletcher

John Suckling

Nikolaus Weiskopf

Pasco Fearon

**Project managers:**

Becky Inkster

Gita Prabhu

**Postdoctoral research associates and associated research fellows:**

Eran Eldar

Tobias Hauser

Konstantinos Ioannidis

Gemma Lewis

Alda Mita

Michael Moutoussis

Sharon Neufeld

Ela Polek-MacDaeid

Rafael Romero-Garcia

Michelle St Clair

Jan Stochl

Roger Tait

Beata Tick

Umar Toseeb

Anne-Laura van Harmelen

Petra Vértes

Kirstie Whitaker

Geert-Jan Will

Gabriel Ziegler

Jorge Zimbron

**PhD & MSc students:**

Joost Haarsma

Sian Davies

Juliet Griffin

Michael Hart

Jakob Seidlitz

Maxwell Shinn

František Váša

Konrad Wagstyl

Data managers:

Cinly Ooi

Barry Widmer

**Research assistants:**

Ayesha Alrumaithi

Sarah Birt

Kalia Cleridou

Hina Dadabhoy

Ashlyn Firkins

Sian Granville

Elizabeth Harding

Alexandra Hopkins

Daniel Isaacs

Janchai King

Clare Knight

Danae Kokorikou

Christina Maurice

Cleo McIntosh

Jessica Memarzia

Harriet Mills

Ciara O'Donnell

Sara Pantaleone

Jennifer Scott

Alison Stribling

**Administration team:**

Junaid Bhatti

Neil Hubbard

Natalia Ilicheva

Michael Kentell

Ben Wallis

Laura Villis
